# Circulating tumor DNA as a therapy response marker in metastatic colorectal cancer

**DOI:** 10.18632/oncoscience.559

**Published:** 2022-06-24

**Authors:** Xiaoju Max Ma, Stephanie J. Yaung

**Affiliations:** ^1^Coherus BioSciences, Redwood City, CA 94065, USA; ^2^Roche Sequencing Solutions Inc., Pleasanton, CA 94588, USA

**Keywords:** ctDNA, colorectal cancer, therapy response, disease monitoring, liquid biopsy

Survival rates of patients with metastatic colorectal cancer (mCRC) have improved in recent years due to an increase in possible treatment options [1]. While chemotherapy remains a treatment backbone for patients with mCRC, other therapies are available in combination with chemotherapy or as later line options, including antibodies against VEGF or EGFR, multikinase inhibitors, and immune checkpoint inhibitors [1]. Therefore, early evaluation of response to treatment is critical to inform appropriate therapeutic management. Monitoring response early and accurately may enable more adaptive and personalized regimens, limit toxicity of ineffective therapies and allow an early switch to potentially more effective treatments. Currently, tumor response is performed routinely with radiological assessments using computed tomography (CT) scans every 2 to 6 months. However, challenges in visualizing small changes in tumor lesions and radiation exposure prevent more frequent imaging strategies.

Liquid biopsies have emerged as a highly sensitive method to non-invasively monitor tumor burden and assess response based on tumor-specific genomic information [2]. In multiple solid tumor types, quantifying changes in the level of circulating tumor DNA (ctDNA) based on tumor-specific mutations can serve as a therapy response marker [2]. Changes in ctDNA can predate those seen on imaging [3]. Several studies have shown that a decrease in ctDNA level, as early as 2 weeks and as late as 8 weeks, after initiation of chemotherapy is associated with longer progression-free survival (PFS) in patients with mCRC [4–8]. Earlier plasma samplings, within the first 3 days following chemotherapy initiation seem too early to be informative of response [4, 9]. Given that later plasma samplings beyond 8 weeks after chemotherapy initiation have been unexplored, we examined how post-induction ctDNA levels may serve as a prognostic biomarker in patients with mCRC following 4–6 month induction therapy [10].

We assessed ctDNA levels as a prognostic marker for PFS in the Sequencing Triplet With Avastin and Maintenance (STEAM; NCT01765582) Trial, which was a randomized, phase II trial investigating efficacy of bevacizumab (BEV) with FOLFOX and 5-fluorouracil/leucovorin/irinotecan (FOLFIRI), administered concurrently or sequentially, versus FOLFOX-BEV in first-line mCRC. There was a 4-month induction phase with the chemotherapy regimen administered in 2-week cycles, and optional extension of induction up to an additional 2 months at the investigator’s discretion. Induction treatment was followed by maintenance with 5-fluorouracil, leucovorin, and bevacizumab every 2 weeks or capecitabine and bevacizumab every 3 weeks.

As part of the exploratory biomarker analyses in STEAM, retrospective sequencing was performed on tumor tissue and plasma with the AVENIO Expanded Kits (for research use only; not for use in diagnostic procedures), which utilizes a hybrid-capture panel targeting 77 genes with known or emerging value as therapeutic biomarkers. Among the 280 patients enrolled in STEAM, 183 had tumor tissue, 118 had matched pre-induction plasma, and 54 had matched post-induction plasma with evaluable sequencing data. Post-induction plasma had to be collected within 60 days of last drug induction. ctDNA levels in plasma were calculated based on somatic single-nucleotide variants pre-defined by the matched tissue sample. While pre-induction levels of ctDNA did not appear to be associated with PFS following induction therapy, we found that lower post-induction ctDNA levels were associated with better PFS (HR = 0.33; 95% CI, 0.17–0.63; log-rank *P* = 0.0005). Furthermore, a 10-fold or 100-fold reduction in ctDNA levels between pre and post-induction plasma was associated with better PFS (HR = 0.24; 95% CI, 0.10–0.60; log-rank *P* = 0.0008; HR = 0.24; 95% CI, 0.11–0.51; log-rank *P* = 0.0001, respectively). These results demonstrate that ctDNA quantification in post-induction plasma may serve as a prognostic biomarker for mCRC post-treatment outcomes.

One limitation of our study is that blood sampling was only performed at pre-induction and post-induction. More serial time points in-between could have provided earlier assessment of ctDNA-based response. In STEAM, the additional 2 month induction period was based on regimen tolerability and CT scan-based tumor assessment (good response defined as CR, PR, or SD). While more studies are required to define a specific time point for routine ctDNA assessment, one can imagine a possible use of ctDNA readout to personalize duration of induction therapy in this setting.

Our results support further investigation of using ctDNA for disease monitoring in mCRC. Taken together with other studies, ctDNA quantification is a robust marker in mCRC that can enable non-invasive assessment of therapy response weeks after initiation of chemotherapy or after completion of induction therapy. ctDNA may also enable disease monitoring in mCRC treated with immunotherapy and multikinase inhibitors [11, 12]. Further work such as prospective trials to demonstrate clinical benefit of changing treatment based on ctDNA information are needed. Nonetheless, it is clear that ctDNA is a promising therapy response marker in mCRC that may help drive future clinical practice to more personalized, post-treatment disease management and enable patient access to more therapy options.

## References

[1] Riedesser JE, Ebert MP, Betge J. Precision medicine for metastatic colorectal cancer in clinical practice. Ther Adv Med Oncol. 2022; 14:17588359211072703. 10.1177/17588359211072703. 35237350 PMC8882813

[2] Boonstra PA, Wind TT, van Kruchten M, Schuuring E, Hospers GAP, van der Wekken AJ, de Groot DJ, Schröder CP, Fehrmann RSN, Reyners AKL. Clinical utility of circulating tumor DNA as a response and follow-up marker in cancer therapy. Cancer Metastasis Rev. 2020; 39:999–1013. 10.1007/s10555-020-09876-9. 32367253 PMC7497299

[3] Diaz LA Jr, Bardelli A. Liquid biopsies: genotyping circulating tumor DNA. J Clin Oncol. 2014; 32:579–86. 10.1200/JCO.2012.45.2011. 24449238 PMC4820760

[4] Tie J, Kinde I, Wang Y, Wong HL, Roebert J, Christie M, Tacey M, Wong R, Singh M, Karapetis CS, Desai J, Tran B, Strausberg RL, et al. Circulating tumor DNA as an early marker of therapeutic response in patients with metastatic colorectal cancer. Ann Oncol. 2015; 26:1715–22. 10.1093/annonc/mdv177. 25851626 PMC4511218

[5] Garlan F, Laurent-Puig P, Sefrioui D, Siauve N, Didelot A, Sarafan-Vasseur N, Michel P, Perkins G, Mulot C, Blons H, Taieb J, Di Fiore F, Taly V, Zaanan A. Early Evaluation of Circulating Tumor DNA as Marker of Therapeutic Efficacy in Metastatic Colorectal Cancer Patients (PLACOL Study). Clin Cancer Res. 2017; 23:5416–25. 10.1158/1078-0432.CCR-16-3155. 28576867

[6] Osumi H, Shinozaki E, Yamaguchi K, Zembutsu H. Early change in circulating tumor DNA as a potential predictor of response to chemotherapy in patients with metastatic colorectal cancer. Sci Rep. 2019; 9:17358. 10.1038/s41598-019-53711-3. 31758080 PMC6874682

[7] Jia N, Sun Z, Gao X, Cheng Y, Zhou Y, Shen C, Chen W, Wang X, Shi R, Li N, Zhou J, Bai C. Serial Monitoring of Circulating Tumor DNA in Patients With Metastatic Colorectal Cancer to Predict the Therapeutic Response. Front Genet. 2019; 10:470. 10.3389/fgene.2019.00470. 31164904 PMC6536571

[8] Parikh AR, Mojtahed A, Schneider JL, Kanter K, Van Seventer EE, Fetter IJ, Thabet A, Fish MG, Teshome B, Fosbenner K, Nadres B, Shahzade HA, Allen JN, et al. Serial ctDNA Monitoring to Predict Response to Systemic Therapy in Metastatic Gastrointestinal Cancers. Clin Cancer Res. 2020; 26:1877–85. 10.1158/1078-0432.CCR-19-3467. 31941831 PMC7165022

[9] Moser T, Waldispuehl-Geigl J, Belic J, Weber S, Zhou Q, Hasenleithner SO, Graf R, Terzic JA, Posch F, Sill H, Lax S, Kashofer K, Hoefler G, et al. On-treatment measurements of circulating tumor DNA during FOLFOX therapy in patients with colorectal cancer. NPJ Precis Oncol. 2020; 4:30. 10.1038/s41698-020-00134-3. 33299124 PMC7666126

[10] Max Ma X, Bendell JC, Hurwitz HI, Ju C, Lee JJ, Lovejoy A, Mancao C, Nicholas A, Price R, Sommer N, Tikoo N, Yao L, Yaung SJ, Palma JF. Disease Monitoring Using Post-induction Circulating Tumor DNA Analysis Following First-Line Therapy in Patients with Metastatic Colorectal Cancer. Clin Cancer Res. 2020; 26:4010–17. 10.1158/1078-0432.CCR-19-1209. 32220893

[11] Kasi PM, Budde G, Krainock M, Aushev VN, Koyen Malashevich A, Malhotra M, Olshan P, Billings PR, Aleshin A. Circulating tumor DNA (ctDNA) serial analysis during progression on PD-1 blockade and later CTLA-4 rescue in patients with mismatch repair deficient metastatic colorectal cancer. J Immunother Cancer. 2022; 10:e003312. 10.1136/jitc-2021-003312. 35101943 PMC8804692

[12] Wang C, Chevalier D, Saluja J, Sandhu J, Lau C, Fakih M. Regorafenib and Nivolumab or Pembrolizumab Combination and Circulating Tumor DNA Response Assessment in Refractory Microsatellite Stable Colorectal Cancer. Oncologist. 2020; 25:e1188–94. 10.1634/theoncologist.2020-0161. 32406541 PMC7418365

